# Platelet Lysate Inhibits NF-κB Activation and Induces Proliferation and an Alert State in Quiescent Human Umbilical Vein Endothelial Cells Retaining Their Differentiation Capability

**DOI:** 10.3390/cells8040331

**Published:** 2019-04-09

**Authors:** Alessio Romaldini, Valentina Ulivi, Marta Nardini, Maddalena Mastrogiacomo, Ranieri Cancedda, Fiorella Descalzi

**Affiliations:** 1 Department of Experimental Medicine, University of Genova, via Alberti 2, 16132 Genova, Italy; alessio.romaldini@gmail.com (A.R.); valentina.ulivi@yahoo.it (V.U.); nardinimarta88@gmail.com (M.N.); descalzi.fiorella@gmail.com (F.D.); 2 Department of Internal Medicine, University of Genova, via Alberti 2, 16132 Genova, Italy; maddalena.mastrogiacomo@unige.it

**Keywords:** endothelial cells, human umbilical vein endothelial cells (HUVEC), platelet lysate (PL), platelet factors, angiogenesis, ERK, AKT, HMGB-1

## Abstract

Injured blood vessel repair and blood circulation re-establishment are crucial events for tissue repair. We investigated in primary cultures of human umbilical vein endothelial cells (HUVEC), the effects of platelet lysate (PL), a cocktail of factors released by activated platelets following blood vessel disruption and involved in the wound-healing process triggering. PL exerted a protective effect on HUVEC in an inflammatory milieu by inhibiting IL-1α-activated NF-κB pathway and by inducing the secretion of PGE_2_, a pro-resolving molecule in the wound microenvironment. Moreover, PL enhanced HUVEC proliferation, without affecting their capability of forming tube-like structures on matrigel, and activated resting quiescent cells to re-enter cell cycle. In agreement with these findings, proliferation-related pathways Akt and ERK_1/2_ were activated. The expression of the cell-cycle activator Cyclin D1 was also enhanced, as well as the expression of the High Mobility Group Box-1 (HMGB1), a protein of the alarmin group involved in tissue homeostasis, repair, and remodeling. These in vitro data suggest a possible in vivo contribution of PL to new vessel formation after a wound by activation of cells resident in vessel walls. Our biochemical study provides a rationale for the clinical use of PL in the treatment of wound healing-related pathologies.

## 1. Introduction

Wound healing is the body’s physiological response to an injury or a disease in order to restore tissue or organ integrity and homeostasis. Adult mammals, including humans, have a limited regenerative potential and tend to repair wounds by fibrosis and scarring. Moreover, fibrotic repair, as result of a chronic inflammation or a prolonged insult, may determine a complex medical case with severe clinical complications [1]. On the contrary, an early and transient inflammatory response represents the critical step for a successful regeneration process [2].

The new frontier of regenerative medicine aims at the development and characterization of advanced therapy medicinal products able to re-activate and to enhance endogenous regeneration pathways, which were lost during evolution and human ontogenesis. In this scenario, platelet-derived products are a promising perspective because they are based on a well-balanced cocktail of more than 300 bioactive factors released by activated platelets following clot formation and platelet degranulation. Physiologically, these factors trigger the tissue regeneration/repair process and are involved in all subsequent steps of the wound healing [3,4]. Therefore, they could be used as a powerful therapeutic tool to trigger and enhance the healing process. Encouraging results were already achieved by the use of different platelet by-products in dental and maxillofacial surgery [5], in orthopedics [6,7], and in ophthalmology [8,9]. Clinical trials, demonstrating beneficial effects for the treatment of degenerative cartilage diseases, were conducted with platelet-rich plasma (PRP) [10] and also with autologous platelet lysate (PL) [11].

In the recent years, we evaluated the activity of PRP and PL on cells potentially involved in the repair/regeneration of several tissues in order to identify the activated pathways leading to tissue healing. We demonstrated an increased proliferation, consequent to a stimulation by platelet-derived factors, in different types of human tissue-resident cells, such as keratinocytes [12], osteoblasts [13], articular chondrocytes [14], and adipose-derived stromal cells [15]. In the investigated cell systems, we observed a strong initial and transient pro-inflammatory activity of PL resulting in NF-κB activation and secretion of pro-inflammatory cytokines [12,13,14,15], the closure of an in vitro scratch wound [12], and a strong activation of quiescent cells, which resumed proliferation keeping the ability to differentiate in permissive conditions [13,16]. However, not only tissue-specific progenitors are activated and take part in the wound-healing process, but also circulating cells, possibly from bone marrow, could be recruited in the wound site [17,18].

In this study, we evaluated the in vitro effects of PL on primary human umbilical vein endothelial cells (HUVEC) because the endothelial cells represent the first cell population responding to blood extravasation and coagulation, platelet activation and degranulation, and the resulting inflammatory milieu. Several studies have reported the effects on endothelial cells of different platelet derivatives, such as PRP [19,20,21], platelet-released supernatant [22,23], or PL [24,25]. We choose to focus our attention on the activity of PL obtained by the lysis of platelets not contaminated by plasmatic molecules in order to specifically investigate the effects of human platelet content on HUVEC and to distinguish between the effects of platelet content and the effects of plasma or serum molecules. To mimic as much as possible the wound microenvironment, as an inflammatory stimulus, we included in our in vitro system, IL-1α, an emerging important factor in the initiation and maintenance of inflammation [26]. The adopted in vitro system allowed examination of the induced endothelial cell responses during the early stages of the wound-healing process. In particular, we investigated the role played by PL on the modulation of the inflammation-related NF-κB pathway in inflammatory conditions induced by IL-1α and on the secretion of cytokines and factors under both physiological and inflammatory conditions. We monitored the proliferation of HUVEC in the presence of PL and we verified the in vitro angiogenic capability of the cells expanded in PL. Finally, we focused on the effect of PL on quiescent HUVEC and we observed the induction of cell proliferation and of proliferation-related pathways, as well as the expression of the High Mobility Group Box-1 (HMGB-1), a protein of the alarmin group involved in homeostasis, repair, and remodeling of tissues. This in vitro biochemical study is aimed at giving a rationale for the current therapeutic use of PL in the treatment of difficult-to-heal wounds.

## 2. Materials and Methods

### 2.1. Materials

Medium 199 with Earle’s Salts, fetal bovine serum (FBS), L-glutamine, penicillin G-streptomycin sulfate and trypsin-EDTA were obtained from Euroclone Life Sciences Division (Milan, Italy). Culture Petri dishes and plates were from Eppendorf S.r.l. (Milan, Italy). Recombinant human FGF-acidic, human FGF-basic, human EGF, and interleukin-1α (IL-1α) were purchased from Peprotech (London, UK). PHAREPA 25000 U.I./5 mL heparin sodium-salt was obtained from PharmaTex Italia (Milan, Italy). Hydrocortisone-water soluble, Bright-Line™ hemacytometer and protease inhibitor cocktail were purchased from Sigma-Aldrich (St. Louis, MO, USA). Corning® Matrigel^®^ Growth Factor Reduced Basement Membrane Matrix was acquired from Corning (Bedford, MA, USA). TransAM™ NF-κB p65 kit was purchased from Active Motif (La Hulpe, Belgium). Human IL-6 Quantikine ELISA Kit and Human IL-8/CXCL8 Quantikine ELISA Kit were from R&D Systems (Minneapolis, MN, USA). Prostaglandin E_2_ ELISA kit was from Cayman Chemical (Ann Arbor, MI, USA). FITC Annexin V Apoptosis Detection Kit I was from BD Biosciences Pharmingen (San Diego, CA, USA).

NuPAGE™ 4–12% Bis-Tris gels were from Invitrogen (Milano, Italy). Amersham™ Protran™ 0.45 µm NC, Amersham™ ECL™ western blotting detection reagents and Amersham™ hyperfilm™ ECL were obtained from GE Healthcare (Buckinghamshire, UK). Antibodies anti-interleukin-8 (IL-8), anti-interleukin-6 (IL-6), anti-Cyclin D1, and anti-Actin were purchased from Santa Cruz Biotechnology Inc. (Dallas, TX, USA). Antibodies anti-phospho-Akt, anti-Akt, anti-phospho-ERK_1/2_, anti-ERK_1/2_, anti-phospho-STAT3 and anti-STAT3 were acquired from Cell Signaling Technology (Danvers, MA, USA). Antibody anti-HMGB1 was from ProteinTech Group Inc. (Chicago, IL, USA).

### 2.2. HUVEC Harvest and Culture

Primary Human Umbilical Vein Endothelial Cells (HUVEC) were obtained from “Centro di Risorse Biologiche” (CRB) of IRCCS Ospedale Policlinico San Martino (Genova, Italy) after obtaining the approval of this study by the institutional ethics committee. The CRB required the written informed consent by every umbilical cord donor. The HUVEC were guaranteed by CRB to be CD31- and CD106-positive (endothelial cell-specific markers) and CD90- and CD45-negative (fibroblast-specific and leukocyte-specific markers, respectively). The cells were seeded at the density of 6.0 × 10^3^ cells/cm^2^ on gelatin-coated 10 cm Petri dishes and cultured in Medium 199 with Earle’s Salts supplemented with 10% (*v*/*v*) FBS, 2 mM l-glutamine, 100 U/mL penicillin G, 100 µg/mL streptomycin sulfate, 100 mg/L heparin, 10 µg/L FGF-acidic, 10 µg/L FGF-basic, 10 µg/L EGF, 1 mg/L hydrocortisone (complete culture medium). Cells were incubated at 37 °C in a humidified atmosphere with 5% CO_2_. Medium was changed 3 times per week and at 80% confluence cells were split 1:2 by treatment with trypsin-EDTA. For the described experiments, HUVEC were used at passages 3 to 6.

### 2.3. Platelet Lysate Preparation

Platelet lysate was produced starting from buffy coat samples derived from the whole blood of healthy donors and considered a waste by Blood Transfusion Centre of IRCCS Ospedale Policlinico San Martino (Genova, Italy). Buffy coats were obtained within the frame of an agreement between Biorigen Srl and IRCCS Ospedale Policlinico San Martino signed on September 2012 and renewed on 2nd February 2017 (“Deliberazione” n.0084). At the time of blood donation, all donors provided a written informed consent for the use of the donated blood for clinical and scientific applications. The buffy coats of 5 to 10 made anonymous donors were pooled for minimizing the variations among donors and centrifuged at low speed. Platelet-rich plasma (PRP) was separated and centrifuged at high speed in order to sediment the platelets. The pellet was washed 3 times with physiological saline (0.9% *w*/*v* NaCl), in order to eliminate possible contaminants from plasma. Platelets were suspended in physiological saline at a concentration of 10 × 10^6^ platelets/µL and the suspension was subjected to 3 freeze/thaw cycles followed by high-speed centrifugation. The supernatant, containing the cocktail of factors released by the platelets (Platelet Lysate, PL), was collected and stored in aliquots at –20 °C until use. Platelet lysate was supplemented to complete culture medium at a final concentration of 5% (*v*/*v*), approximately corresponding to the highest physiological concentration of platelets in the human blood, without addition of heparin. Three different preparations from different pools of buffy coats were used in the study.

### 2.4. Proliferation Assays

(1) Crystal violet assay: HUVEC were seeded at the density of 6.5 × 10^3^ cells/cm^2^ on gelatin-coated 96-well plate and incubated in complete culture medium for 24 h to enable cell adhesion. The next day, the medium was replaced with complete medium not supplemented (control cells) or supplemented with 5% PL (treated cells). The assay was performed in quintuplicate for each culture condition after 0, 2, 4, and 6 days of PL stimulation, following the protocol described by Nguyen et al. [16]. Three independent experiments were performed on different single-donor HUVEC cultures. The final results are expressed as mean ± SD.
(2) Cell count: HUVEC were seeded on gelatin-coated 24-well plate and cultured in complete culture medium until reaching confluence. The medium was then replaced with complete medium supplemented with 5% PL (treated culture) or not supplemented (control culture). At 0, 3, 6, and 10 days of PL stimulation, cell density was monitored by cell counting using a Bright-Line™ Hemacytometer with an improved Neubauer chamber. For each culture condition, the final result is the n-fold increase of cell density with respect to day 0, expressed as mean ± SD of 3 independent experiments performed in triplicate on different single-donor HUVEC cultures.

### 2.5. Apoptosis Assay

To evaluate the cell apoptotic status after PL treatment, the FITC Annexin V Apoptosis Detection Kit I (BD Biosciences) was used. HUVEC were seeded in complete culture medium at a density of 6.5 × 10^3^ cells/cm^2^ on gelatin-coated 60 mm Petri dish. The next day, the medium was replaced with complete medium not supplemented (control cells) or supplemented with 5% PL (treated cells). After 6 days of treatment, the cells were detached and assayed according to the manufacturer’s instructions. Samples were run on CyAN ADP flow cytometer (Beckman-Coulter, Pasadena, CA, USA) and analyzed with FlowJo 10.0.7 software (FlowJo, LCC, Ashland, OR, USA). For each culture condition, 3 independent experiments were performed.

### 2.6. Tube-Like Structure Formation Assay

Proliferating HUVEC were cultured in complete culture medium un-supplemented (control) or supplemented with 5% PL for a week. The cells were then trypsinized, re-suspended in serum-free medium (no supplements), and seeded at the density of 7 × 10^4^ cells/well on matrigel-coated 24-well plate. Images were taken after 6 h incubation at 37 °C in a humidified atmosphere with 5% CO_2_. Two experiments in duplicate were performed.

### 2.7. Western Blot

To analyze the cytokine production in cell culture media, sub-confluent HUVEC were treated for 1 or 24 h with complete culture medium supplemented with: (i) 5% PL; (ii) 100 U/mL IL-1α; (iii) 5% PL + 100 U/mL IL-1α; and (iv) without any supplement. Cells were then extensively washed with PBS to remove residual factors and incubated in serum-free medium (medium 199 with Earle’s Salts only supplemented with 2 mM l-glutamine, 100 U/mL penicillin G and 100 µg/mL streptomycin sulfate) for 24 h. The different media were collected, clarified at 2000 rpm for 10 min at room temperature and stored at −20 °C. To investigate the PL effect on proliferation-related pathways and on cell-cycle activation, sub-confluent HUVEC were treated with complete culture medium supplemented with 5% PL for multiple time intervals, washed with PBS and lysed by incubating the cell layers on ice for 5 min with an ice-cold buffer containing 50 mM Tris HCl pH 7.5, 150 mM NaCl, 1% (*w*/*v*) sodium deoxycholate, 1% (*v*/*v*) Triton X-100, 0.1% (*w*/*v*) sodium dodecyl sulfate, 0.2% (*w*/*v*) sodium azide and protease inhibitor cocktail. Cell lysates were harvested with cell scrapers, clarified at 10,000 rpm for 15 min at 4 °C and stored at –20 °C. The protein content of both conditioned media and cell lysates was quantified by Bradford protein assay [27].

Electrophoresis was performed in reducing conditions using 25–60 μg of protein loaded on a NuPAGE™ 4–12% Bis-Tris gel and blot was performed as described by Ulivi et al. [28] For each considered marker, a western blot was performed for at least 3 independent experiments corresponding to different single-donor primary HUVEC cultures. Densitometric absorbance was determined by scanning the film and quantifying the band densities using ImageJ software (https://imagej.nih.gov/ij/download.html). The reported results are the average of at least 3 independent experiments ± SD values.

### 2.8. NF-κB Activity Assay

To evaluate the nuclear factor-κB (NF-κB) activity, the TransAM™ NF-κB p65 kit was used. Sub-confluent HUVEC were treated for 1 h or 16 h with complete culture medium supplemented with: (i) 5% PL; (ii) 100 U/mL IL-1α; (iii) 5% PL + 100 U/mL IL-1α; and (iv) without any supplement (control). Media were removed and cells washed with PBS. Whole-cell extracts were prepared and assayed following manufacturer’s instructions. Specificity of the assay was checked by adding soluble wild-type and mutated consensus oligonucleotides acting as competitors for NF-κB binding. For the reported representative experiment, results are expressed as the absorbance values measured in the presence of the mutated oligonucleotide minus those measured in the presence of the wild-type oligonucleotide. This assay was performed in triplicate on 3 independent experiments corresponding to different single-donor primary HUVEC cultures. For each stimulation time, the n-fold increase over control of NF-κB activity induced by IL-1α stimulation and the percentage value of NF-κB activity induced by PL + IL-1α treatment with respect to IL-1α net increase are reported (means ± SD).

### 2.9. IL-8 and IL-6 Quantification

To quantify the IL-8 and IL-6 secretion, the Human IL-8/CXCL8 Quantikine ELISA Kit and the Human IL-6 Quantikine ELISA Kit (R&D systems) were used, respectively. HUVEC were treated for 24h with complete culture medium supplemented with: (i) 5% PL; (ii) 100 U/mL IL-1α; (iii) 5% PL + 100 U/mL IL-1α; and (iv) without any supplement (control). Cells were then extensively washed with PBS and incubated in serum-free medium for 24 h. The different conditioned media were collected and assayed following manufacturer’s instructions. For each conditioned medium, the cytokine secretion is expressed as total protein-normalized mean ± SD of 4 independent experiments performed in duplicate on different single-donor HUVEC cultures.

### 2.10. PGE_2_ Quantification

To quantify the PGE_2_ production, the Prostaglandin E_2_ ELISA kit was used. HUVEC were treated for 24 h with complete culture medium supplemented with: (i) 5% PL; (ii) 100 U/mL IL-1α; (iii) 5% PL + 100 U/mL IL-1α; (iv) without any supplement (control). Cells were then extensively washed with PBS for removing residual factors and incubated in serum-free medium for 24 h. The different conditioned media were collected and assayed following manufacturer’s instructions. Results are expressed as fold change with respect to control. Four determinations were performed in duplicate on 3 different single-donor primary HUVEC cultures.

### 2.11. Statistical Analysis

All data are presented as means and standard deviations based on independent experiments performed on at least three different primary HUVEC cultures, each of them derived from a single donor. The statistical analysis was performed using the paired *t*-Test for NF-κB activity and proliferation assays or using the ordinary one-way ANOVA for IL-8, IL-6, HMGB1, Cyclin D1 and phospho-STAT3 densitometric analysis and PGE_2_ quantification. If ANOVA detected statistically significant differences within the data set, Tukey’s or Dunnett’s multiple comparison tests were used to calculate the significant differences for IL-8 and IL-6 densitometric analysis and PGE_2_ quantification or for HMGB1, Cyclin D1, and phospho-STAT3 densitometric analysis, respectively. All tests were run setting a confidence interval of 95%. 

## 3. Results

### 3.1. PL Down-Regulated NF-κB Pathway in IL-1α-Stimulated Cells

Having in mind that bioactive molecules released by platelets trigger the wound healing and that this process takes place in an inflammatory microenvironment, we focused our attention on the response of endothelial cells to PL in both normal conditions and in the presence of an inflammatory stimulus. In our in vitro system, among the possible pro-inflammatory molecules we tested (i.e., TNFα and IL-1α), we chose IL-1α because in preliminary experiments it showed the best inflammatory response and it is considered an emerging important factor in the initiation and maintenance of inflammation.

**Figure 1:**
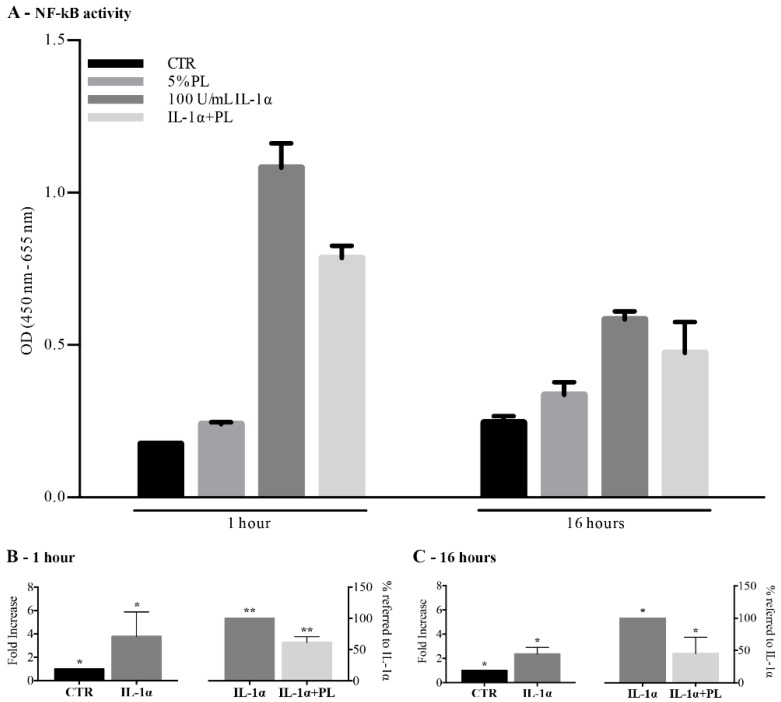
Modulation of NF-κB pathway in human umbilical vein endothelial cells (HUVEC) treated with platelet lysate (PL) under physiological and inflammatory conditions. Sub-confluent HUVEC were treated for 1 h or 16 h with complete medium supplemented with: (i) 5% PL; (ii) 100 U/mL IL-1α; (iii) 5% PL+100 U/mL IL-1α; (iv) without any supplement (control medium, CTR). Whole-cell extracts were analyzed by ELISA-based TransAM™ NF-κB p65 kit. (**A**) Absorbance values of NF-κB activity after 1 h and 16 h stimulation in a representative experiment. (**B**,**C**) NF-κB activity after 1 h (**B**) and 16 h (**C**) exposure to IL-1α, expressed as fold increase over control (left columns), and percentage of activity measured after PL + IL-1α stimulation (right columns) with respect to the IL-1α-induced net increase (100%, correspond to the measured increase of activity due to the IL-1α stimulation i.e., difference between values of stimulated and un-stimulated control cells). For each condition, the average of 3 independent experiments (mean ± SD) assayed in triplicate on different single-donor primary cultures is reported. For 1h stimulation, * and ** symbols refer to *p* = 0.03 and *p* = 0.009, respectively. For 16 h stimulation, * refers to *p* ≤ 0.03.

In particular, we evaluated the activation of NF-κB pathway, a key player in the inflammatory phase response [29], in sub-confluent HUVEC maintained for 1h or 16h in complete culture medium supplemented with: (i) 5% PL; (ii) 100 U/mL IL-1α; and (iii) 5% PL+100 U/mL IL-1α or maintained in un-supplemented control medium (CTR). NF-κB activity determined in a representative experiment where cells were exposed to different culture conditions is presented in Figure 1, Panel A. Panels B shows the fold increase over control when the cells were induced by 1h stimulation with IL-1α and the percentage reduction in the IL-1α-induced NF-κB activity when the culture was supplemented also with PL. Panel C shows the fold increase and the percentage reduction after 16h stimulation of the cells. Values are reported as average values ± SD values of 3 independent experiments. The NF-κB-activity was significantly enhanced by the exposure to IL-1α with respect to control after both 1h and 16h cell stimulation (*p* = 0.03 and *p* = 0.01, respectively) while significantly decreased in cells treated with PL + IL-1α with respect to the IL-1α-treated cells after both 1 h and 16 h stimulation (*p* = 0.009 and *p* = 0.03, respectively). These results indicate an anti-inflammatory activity of PL on HUVEC both at early and late times. 

**Figure 2:**
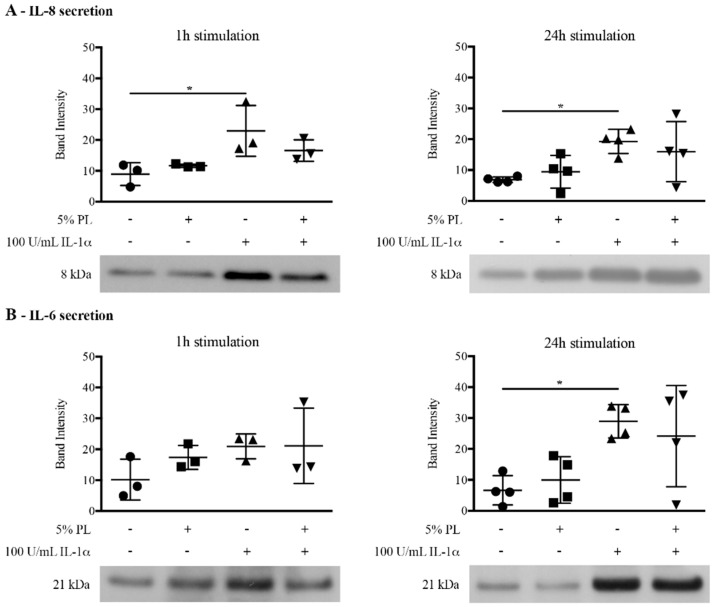
Pro-inflammatory cytokine secretion by HUVEC upon PL stimulation under physiological and inflammatory conditions. HUVEC were treated for 1 h or 24 h with complete medium supplemented with: (i) 5% PL; (ii) 100 U/mL IL-1α; (iii) 5% PL + 100 U/mL IL-1α; and (iv) without any supplement (control medium, CTR). At the end of the stimulation, the media were removed and replaced with serum-free medium. After an additional 24 h incubation, the conditioned media were collected. A western blot analysis of conditioned media was performed to determine the amount of secreted IL-8 (**A**) and IL-6 (**B**). The densitometric analysis of western blots was performed on 3 and 4 independent single-donor primary cultures (means ± SD) for 1 h or 24 h treatment, respectively. The * symbol represents significant differences with *p* ≤ 0.05. Representative western blots are shown under the densitometric analysis.

Considering the negative regulation of NF-κB pathway by PL in an inflammatory milieu, we evaluated the production of two pro-inflammatory cytokines, IL-8 and IL-6, following 1 h and 24 h stimulations with PL under both physiological and inflammatory conditions. By western blot analysis of conditioned media, in PL + IL-1α-treated cells we observed a trend, but we could not detect a significant decrease in the secretion of the pro-inflammatory cytokines induced by IL-1α (Figure 2A,B). Similarly, the ELISA quantification of IL-8 and IL-6 in the 24 h-conditioned media could not reveal any significant difference in the secretion by PL + IL-1α- and IL-1α-treated cells of both IL-8 and IL-6 (data not shown).

### 3.2. PL Increased PGE_2_ Secretion by HUVEC in IL-1α Stimulated Cells

**Figure 3:**
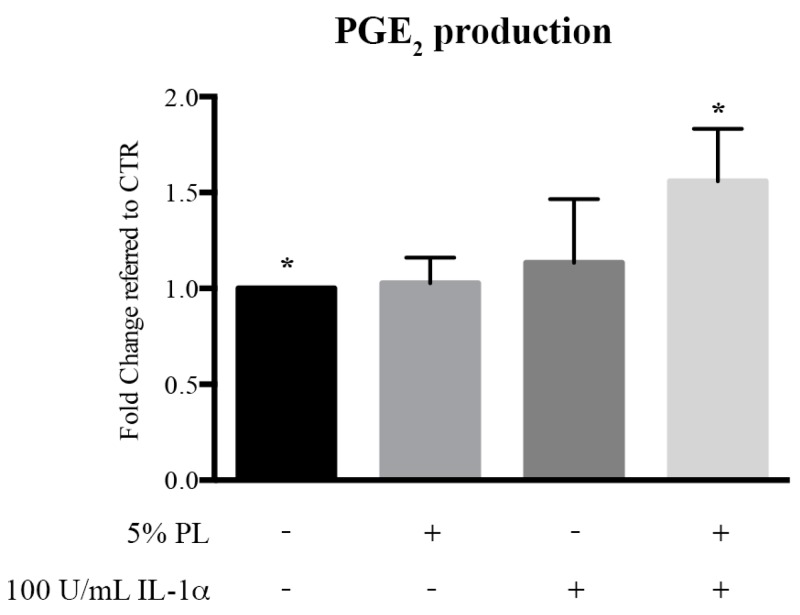
PGE_2_ secretion by HUVEC upon PL stimulation under physiological and inflammatory conditions. HUVEC were treated for 24 h with complete medium supplemented with: (i) 5% PL; (ii) 100 U/mL IL-1α; (iii) 5% PL + 100 U/mL IL-1α; and (iv) without any supplement (control medium, CTR). At the end of the stimulation, the media were removed and replaced with serum-free medium. After additional 24 h incubation, the conditioned media were collected and analyzed by Prostaglandin E_2_ ELISA kit. For each condition, PGE_2_ production is expressed as fold increase with respect to CTR. The average values and relative standard deviation values of 4 determinations performed in duplicate on 3 different single-donor primary cultures are presented. The * symbol represents a significant difference with *p* = 0.02.

In a previous publication, we demonstrated a pro-resolving activity of Platelet Rich Plasma on cells of the immune system to create an anti-inflammatory microenvironment consequent to a PGE_2_ production [30]. A quantitation of PGE_2_ released by HUVEC in the presence of PL under physiological and inflammatory conditions was performed in order to detect a possible protective activity by stimulated endothelial cells at the wound site. We observed a significant increase of PGE_2_ secretion in PL + IL-1α-treated cells with respect to the control (*p* = 0.02; Figure 3), indicating that PL-activated HUVEC could contribute to the resolution of tissue inflammation also by a paracrine mechanism.

### 3.3. PL Enhanced Proliferation of HUVEC Retaining Their Differentiation Capability

**Figure 4:**
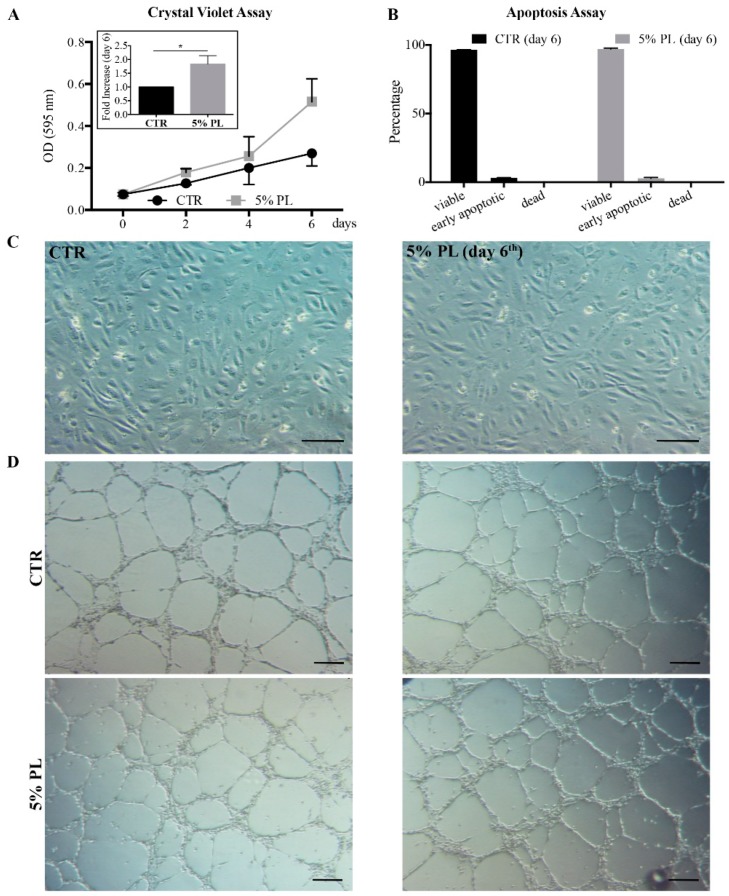
PL effect on proliferation, morphology and angiogenic potential of HUVEC. (**A**) Proliferation of HUVEC, treated with 5% PL supplemented or un-supplemented (control, CTR) complete medium, evaluated at different times by crystal violet staining assay. The average values ± SD of 3 independent experiments performed in quintuplicate on different single-donor primary cultures are reported (insert) Fold increase after 6 days of PL treatment over control (CTR) expressed as mean ± SD of 3 independent experiments. The * symbol refers to *p* = 0.01; (**B**) Percentage of viable, apoptotic and dead cells after 6 days culture in the same condition as in panel A. Apoptosis assay was performed taking advantage of the FITC Annexin V Apoptosis Detection Kit I; (**C**) Morphology of HUVEC cultured in complete medium un-supplemented (control cells, CTR) or supplemented with 5% PL for 6 days. Scale bar = 150 μm; (**D**) Tube-like structure formation assay performed on matrigel using HUVEC grown in complete medium un-supplemented (CTR) or supplemented with 5% PL for 7 days. The assay was performed in the absence of PL. Two pictures related to different positions in the well are reported for each condition. Scale bar = 250 μm.

The PL effect on HUVEC viability and proliferation was evaluated at different times from the PL addition on 3 independent primary cultures. Cells treated with the complete culture medium supplemented with 5% PL had a higher proliferation rate with respect to control cells maintained in complete medium with no supplement, with a significant (1.8 ± 0.3)-fold increase induced by PL at day 6 with respect to the control (*p* = 0.01; Figure 4A). It is important to note that cells grown in the presence of PL maintained an almost 100% viability without any difference with respect to control cells (Figure 4B). We did not observe any major change in the cell morphology between PL-treated cells and control cells (Figure 4C). Moreover, cells grown in PL for 1 week kept the ability to form capillary-like structures when seeded on matrigel and we did not observe qualitative differences between cells expanded in PL and control cells grown in the absence of PL (Figure 4D).

### 3.4. PL Induced Proliferation and an Alert State in Quiescent HUVEC

**Figure 5:**
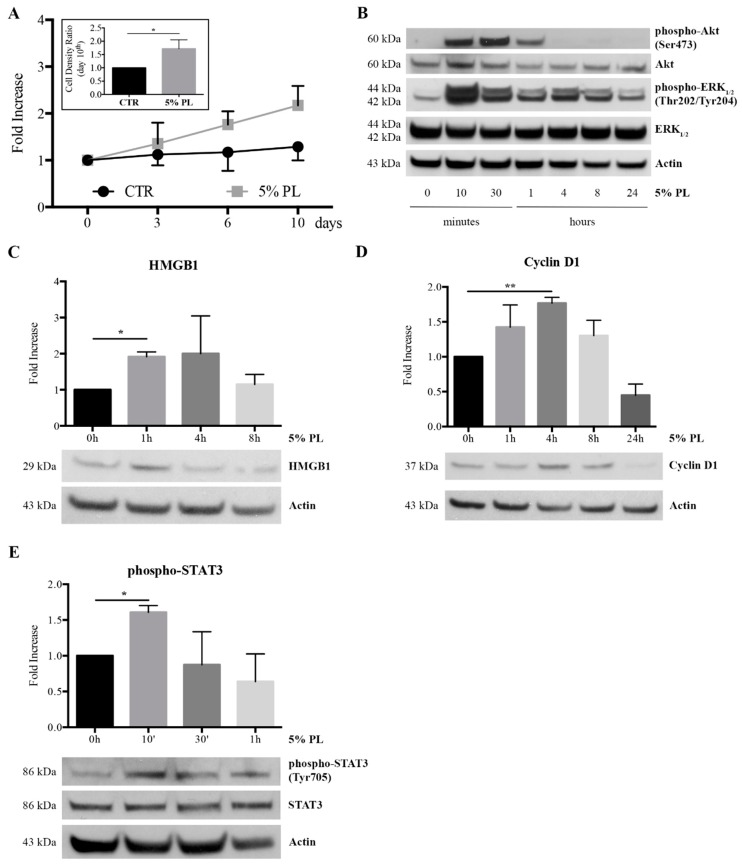
Proliferation of quiescent confluent HUVEC in the presence of PL, modulation of Akt and ERK_1/2_ pathways and activation of HMGB1, cell cycle and STAT3 by PL. (**A**) Proliferation of confluent HUVEC maintained in complete medium (CTR) or treated with complete medium supplemented with 5% PL monitored by cell counting. The values are expressed as fold increase related to the initial time (day 0). The average values ± SD of 3 determinations performed in triplicate on 3 different single-donor primary HUVEC cultures are shown. In the insert, the cell density ratio between PL-treated and control cells (CTR) at day 10th of PL treatment is reported. Ratio was separately calculated in 3 independent experiments and expressed as mean ± SD. The * symbol refers to *p* = 0.05. (**B**) Western blot analysis of HUVEC treated with complete medium supplemented with 5% PL for different times. Cell lysates were analyzed by western blot with antibodies raised against phospho-Akt, Akt, phospho-ERK_1/2_, ERK_1/2_ and Actin. The Akt, ERK_1/2_, and Actin were used as internal controls. Three independent experiments performed on different primary HUVEC cultures yielded the same results. (**C**) Western blot analysis of cells treated with complete medium supplemented with 5% PL for different times using antibody raised against HMGB1. In the upper panel, the densitometric analysis of HMGB1-probed blots performed on 3 independent single-donor primary cultures is reported as fold increase referred to 0h (means ± SD). The * symbol represents significant difference with *p* = 0.01. Under the column panel, a representative western blot for HMGB1 is shown. Actin was blotted as internal control. (**D**) The PL effect on cell cycle of cells treated with 5% PL in complete medium for different times. A western blot analysis of the cell lysates was performed to evaluate the Cyclin D1 expression. In the upper panel, the densitometric analysis of Cyclin D1-probed blots performed on 3 independent single-donor primary cultures is reported as fold increase referred to 0 h (means ± SD). The ** symbol represents a significant difference with *p* = 0.002. Under the column panel, a representative western blot for Cyclin D1 is shown. Actin was blotted as internal control. (**E**) Western blot analysis for the activation of angiogenesis-related STAT3 by PL in cells treated with complete medium supplemented with 5% PL for different times. Cell lysates were analyzed using antibody raised against phospho-STAT3. In the upper panel, the densitometric analysis of pospho-STAT3-probed blots performed on 3 independent single-donor primary cultures is reported as fold increase referred to 0h (means ± SD Each phospho-STAT value was related to and corrected for STAT value. The significant difference 0 h versus 10’ corresponds to *p* = 0.02 (*).

Considering the enhancement of proliferation observed in HUVEC cultures in response to the PL stimulation and our previous studies demonstrating a PL-induced activation of quiescent human osteoblasts and articular chondrocytes [13,16], we cultured HUVEC in complete culture medium to confluence and we treated the confluent growth-arrested cells with complete medium supplemented with 5% PL or not supplemented (control) for 10 days. We monitored cell growth by cell counting. The PL induced a proliferation resumption by confluent HUVEC that reached a higher (1.7 ± 0.3)-fold cell density than control cells at day 10th (*p* = 0.05, Figure 5A). For each experimental condition, we report values expressed as fold increase with respect to the initial time (day 0). Moreover, we investigated the modulation of proliferation-related Akt and ERK_1/2_ pathways [31,32] and the expression of the cell-cycle associated Cyclin D1. Cell lysates were collected at different times from the PL addition and analyzed by western blot. In agreement with the observed resumption of cell proliferation, the activation of both Akt and ERK_1/2_ pathways was observed already 10 min after the exposure to PL followed by a progressive decrease, more rapid for Akt than for ERK (Figure 5B). Three independent experiments performed on different primary HUVEC cultures yielded the same results. The Cyclin D1 expression was enhanced already after 1 h of PL treatment reaching its maximum level at 4 h (*p* = 0.002 for 0 h versus 4 h) and decreasing at later times (Figure 5D). 

We also considered the possible effect of PL in inducing the synthesis of HMGB1, one of the best characterized members of the alarmin family which are produced by injured, or activated cells, and make cells at the lesion site more susceptible to factors released in wound thus promoting their proliferation [33,34]. Interestingly, HMGB1 was significantly increased after 1-h stimulation (*p* = 0.01 for 0 h versus 1 h), decreasing at later times (Figure 5C). In agreement with a possible implication of PL in restoring blood vasculature in wounded tissues, we also found a very rapid and transient activation of STAT3 (*p* = 0.02 for 0 h versus 10’, Figure 5E), which is a critical transcription factor in angiogenesis [35]. 

## 4. Discussion

Injured blood vessel restoration and blood circulation re-establishment are crucial events for tissue repair. Our work focused on the behavior of endothelial cells in a wound-like microenvironment characterized by the presence of an inflammatory stimulus and by the transition from plasma to serum, i.e., from plasma to a mixture of plasma proteins depleted of coagulation factors and also including growth factors released by activated platelets. The new microenvironment plays a major role in restoring tissue function, but, at the end of the healing process, when the blood circulation is re-established in the newly formed tissue, cells are again exposed to plasma. Indeed, in vivo, the plasma to serum transition is needed for initiating proper healing, but, in the newly formed tissue, serum must be replaced by plasma for the reactivation of tissue functions [36]. 

By using an in vitro model, in this paper, we showed how platelet lysate (PL), used at a concentration approximately corresponding to the highest physiological concentration of platelets in the human blood, could modify the behavior of endothelial cells and, hence, play a significant regulatory role in the different phases of tissue healing:

### 4.1. PL Favored the Resolution of Inflammation in Endothelial Cells by Inhibiting IL-1α-Activated NF-κB Pathway

Physiologically, after an injury, platelets leak from damaged blood vessels and the inflammatory phase is initiated by the platelet degranulation leading to the release of growth factors acting as key players in the tissue healing process. The inflammatory phase is characterized by the clearance of microbial contamination and the removal of devitalized tissue by migrating macrophages. We previously investigated the effect of PL in an inflammatory milieu in several cell systems and we demonstrated an early and transiently enhanced activation of the pro-inflammatory NF-κB pathway induced by IL-1α in human keratinocytes, osteoblasts, articular chondrocytes, and murine bone marrow-derived stromal cells [12,13,14,37]. This early activation was paralleled by an early increased secretion of pro-inflammatory cytokines IL-6 and IL-8 and, in keratinocytes and chondrocytes, also by an increased production of the antimicrobial lipocalin NGAL. In human articular chondrocytes, this effect was transient because, after the early inflammatory burst, PL inhibited the activation of NF-κB, induced by IL-1α [14]. This finding was in agreement with other reports that showed a pro-resolving activity of PL and/or PRP [10]. We believe that platelet-released factors exerts an immediate pro-inflammatory effect causing an immediate antimicrobial response by the tissue that releases antimicrobial proteins such as NGAL [12,14], and the migration of neutrophils and macrophages [38] that engulf contaminant microorganisms and remove the devitalized tissue. At later time, these factors exert an opposite effect by inhibiting NF-κB activation and promoting resolution of the inflammatory phase. 

Given that endothelial cells are the first cells in contact with PL after vessel injury, we treated HUVEC with PL for different times both in the presence and in the absence of IL-1α. IL-1α is not the only pro-inflammatory factor present at the wound site because also other cytokines are locally increased. However, after we performed some preliminary experiments also with TNFα, we selected IL-1α as the best pro-inflammatory agent for our purpose.

The PL inhibited the activation of NF-κB induced by IL-1α already after 1-h treatment as well as after 16-h treatment showing an anti-inflammatory activity at both the early and the late considered times. This finding is in agreement with the anti-inflammatory activity of PRP described in literature [39] and provides a possible rationale to this effect. We also observed that, at variance with the other so far investigated cell systems, PL did not significantly enhance the production of IL-6 and IL-8 induced by IL-1α, nor induced a significant repression of these two cytokines. The response of the different assayed primary cultures was quite variable as demonstrated by the standard deviation. and a slight decrease in their secretion could possibly be observed as a trend only in the western blot analysis. Taken together, these data suggest that, already immediately after the injury, endothelial cells are protected by PL in the inflammatory milieu of the wound. 

### 4.2. PL Increased PGE_2_ Secretion by IL-1α Treated Endothelial Cells

A significant increase of PGE_2_ secretion by HUVEC was observed following PL addition in inflammatory conditions indicating that PL-activated HUVEC could possibly contribute to the resolution of tissue inflammation in the wound microenvironment by acting on inflammatory cells. Indeed, an anti-inflammatory activity of PGE_2_ was demonstrated toward macrophages, inducing the functional switch from M1 inflammatory phenotype to M2 pro-resolving phenotype [40]. In a previous publication from our laboratory, it was already demonstrated a pro-resolving activity of platelet rich plasma on cells of the immune system to create an anti-inflammatory microenvironment related to PGE_2_ production [30]. The presented results provide an indication of an anti-inflammatory activity within the wound microenvironment, driven by PGE_2_ produced by endothelial cells in response to PL at an early time.

### 4.3. PL Enhanced Proliferation of Endothelial Cells without Affecting Their Differentiation Capability

Our goal was to dissect the system and to specifically investigate the effect of human platelet-contained molecules on endothelial cells maintained in standard culture conditions, i.e., in the presence of fetal bovine serum. PL enhanced the proliferation of HUVEC, without affecting their viability and capability of forming tube-like structures on matrigel. Our findings are in agreement with already published data showing that platelet-released molecules support proliferation of cells from different tissues, including endothelial cells, which present a significantly lower percent of apoptotic cells and increased proliferation rates [41]. The maintained capacity of HUVEC expanded in PL to form tube-like structures on matrigel is in agreement with Tasev et al. [42], although, in that study, the platelet lysate used as serum substitute was made in human plasma. 

### 4.4. PL Induced Proliferation of Quiescent HUVEC

Cellular quiescence is a non-proliferating condition of the cells at a stage of basic metabolism. Indeed, the cells of the body are mainly non-dividing cells and can be schematically classified in irreversibly arrested cells (senescent or terminally differentiated) and quiescent cells, able to re-enter the proliferative cell cycle in response to physiological growth signals. Quiescent cells include many adult stem cells, tissue progenitor cells, and possibly differentiated cells. The reactivation of quiescent cells leading to their proliferation is the crucial event triggering tissue repair and regeneration (for a review see [43]). We previously reported that quiescent cultured osteoblasts exposed to PL resumed proliferation and retain their differentiation capability [13]. We also recently reported that human chondrocytes exposed to PL in a quiescent stage re-enter the cell cycle and proliferate [16]. In the present study, we observed that resting confluent HUVEC in complete culture medium, a condition mimicking the physiological quiescence, were activated by PL and resumed cell proliferation up to a cell concentration approximately double than the one of not PL-stimulated, control cells. We believe that this is an important novel finding not reported in previous publications reporting PL induction of endothelial cell proliferation [24,25].

Pathways involved in cell activation and proliferation of quiescent cells were also investigated. The role of ERKs was described in PL driven endothelial cell repair in a model of scratch wound [23]. Moreover, in a previous publication, we demonstrated that ERKs and AKT were activated by PL in quiescent osteoblasts and that these factors were responsible for the cell proliferation since specific inhibitors of the two pathways suppressed proliferation [13]. Here we report that, also in quiescent endothelial cells the proliferation related ERKs and AKT were phosphorylated and the expression of the cell-cycle activator Cyclin D1 was enhanced following the cell treatment with PL.

An early enhancement of HMGB1 was also observed. HMGB1 is the best characterized factor of the alarmin family, a group of endogenous molecules released from injured or activated cells first described as molecules inducing immune/inflammatory response at the site of injury, but subsequently shown to be also involved in tissue homeostasis including repair and remodeling of different tissues [44,45]. A recent extensive study described how the molecule could induce inflammation and/or regeneration of the wounded tissue according to the different redox forms that act on distinct receptors [44]. Our finding that PL timely induces HMGB1 in endothelial cells provides an additional information on PL activity and opens a field of further investigation about the possible secretion and autocrine activity of HMGB1 in endothelial cells activation and proliferation. 

HUVEC and HAEC (human aortic endothelial cells) derived from vessel walls, are considered differentiated endothelial cells, also containing a complete hierarchy of endothelial progenitor cells (EPCs). Indeed, a diversity of EPCs exists in human vessel playing an important role in maintaining vessel integrity [46,47]. We demonstrated that resting cultured confluent HUVEC are activated and resume proliferation following a PL treatment suggesting that an activation of the resident cell population, possibly progenitors and differentiated cells, could occur also in vivo after an injury.

Several studies reported that EPCs could be isolated also from adult peripheral and umbilical cord blood. These progenitors are thought to originate from bone marrow, to circulate in the peripheral blood and to be involved in neovascularization and wound healing [48,49]. However, mechanisms for vessel repair in wound are not currently well defined. Cells from bone marrow are recruited to the site of injury by migration trough the blood circulation, whereas cell activation and recruitment from vessel walls occurs via direct activation of local cells. We believe that, in the process of injury repair, cells recruited from bone marrow and resident cells contribute to new vessel formation in an extent that may vary depending on the type of injury and possibly on the tissue type involved. 

In this scenario, we observed that PL was also able to induce an early and transient activation of the STAT3 pathway in agreement with reports indicating that STAT3 signaling is important and necessary for endothelial cell proliferation, migration, and microvascular tube formation [35] and also for in vivo angiogenesis induction [50]. 

## 5. Conclusions

In this paper, we demonstrated a beneficial activity of PL treatment on HUVEC resulting in the inhibition of the inflammatory response, the enhancement or the resumption of proliferation of endothelial cells, all retaining the differentiation capability, concomitant with the activation of proliferation-related pathways, the induction of the synthesis of the alarmin HMGB1, and the activation of angiogenesis-related STAT3 pathway, thus providing a biochemical approach aimed at giving a rationale for the current therapeutic use of PL in the treatment of wound healing-related pathologies.
